# Is there a correlation between plasma phosphorylated tau concentration and advancing age? A narrative review

**DOI:** 10.1002/alz70856_102008

**Published:** 2025-12-25

**Authors:** Jemma Hazan, Kathy Y Liu, Robert Howard, Henrik Zetterberg, Nick C Fox

**Affiliations:** ^1^ University College London, London, London, United Kingdom; ^2^ Division of Psychiatry, University College London, London, London, United Kingdom; ^3^ Division of Psychiatry, University College London, London, United Kingdom; ^4^ Department of Psychiatry and Neurochemistry, Institute of Neuroscience and Physiology, the Sahlgrenska Academy, University of Gothenburg, Molndal, Sweden; ^5^ Wisconsin Alzheimer's Disease Research Center, University of Wisconsin‐Madison, School of Medicine and Public Health, Madison, WI, USA; ^6^ University College London, London, United Kingdom, London, United Kingdom; ^7^ Hong Kong Center for Neurodegenerative Diseases, Hong Kong, Hong Kong, China; ^8^ UK Dementia Research Institute at UCL, London, United Kingdom; ^9^ Dementia Research Centre, UCL Queen Square Institute of Neurology, London, United Kingdom; ^10^ UK Dementia Research Institute, Queen Square Institute of Neurology, University College London, London, United Kingdom

## Abstract

**Background:**

Plasma phosphorylated tau (*p*‐tau) protein levels are elevated in Alzheimer's disease when compared to cognitively unimpaired individuals. They represent potential candidate blood biomarkers for use in memory services where CSF examination is not available. However, the effect of age on plasma *p*‐tau levels remains undetermined. Limited studies have investigated the correlation between age and plasma *p*‐tau thus far, and fewer still have discriminated by brain amyloid pathology status. Characterising such a correlation and determining if this is influenced by sociodemographic factors or medical comorbidities is important for establishing blood biomarker reference ranges.

**Method:**

This review summarises the available evidence regarding the correlation of age with plasma *p*‐tau levels and uses ADNI data to estimate fixed effects of age, creatinine, baseline BMI, sex, and race on plasma *p*‐tau181 concentration.

**Result:**

From analysis of the ADNI data, there was a positive significant correlation between *p*‐tau181 levels and age, creatinine, and minority ethnic group, and a significant negative correlation between baseline BMI and *p*‐tau181 levels, when using the entire sample data. There was no between group (AD vs control) difference in the relationship of *p*‐tau181 levels with age, but individuals with AD showed a more negative association between ptau181 and creatinine compared to controls. We found that the positive correlation between age and *p*‐tau181 levels was not accounted for by higher creatinine levels, BMI and race.

**Conclusion:**

We provide recommendations to address knowledge gaps for future studies.

